# Safety and effectiveness of bubble continuous positive airway pressure as respiratory support for bronchiolitis in a pediatric ward

**DOI:** 10.1007/s00431-022-04616-3

**Published:** 2022-09-21

**Authors:** Marta Agüera, Maria Melé-Casas, Maria Mercedes Molina, Martí Pons-Odena, Mariona F. de-Sevilla, Juan-José García-García, Cristian Launes, Laura Monfort

**Affiliations:** 1 Paediatrics Department, Hospital Sant Joan de Déu, P. Sant Joan de Déu, no. 2, 08950 Esplugues de Llobregat, Barcelona, Spain; 2 Nurse of Paediatrics, Hospital Sant Joan de Déu, P. Sant Joan de Déu, no. 2, 08950 Esplugues de Llobregat, Barcelona, Spain; 3 Immunological and Respiratory Disorders, Paediatric Critical Care Unit Research Group, Institut de Recerca Sant Joan de Déu, Santa Rosa, no. 39-57, 08950 Esplugues de Llobregat, Spain; 4 Intensive Care Unit Department, Hospital Sant Joan de Déu, P. Sant Joan de Déu, no. 2, 08950 Esplugues de Llobregat, Barcelona, Spain; 5 Paediatric Infectious Diseases Research Group, Institut de Recerca Sant Joan de Déu, Santa Rosa, no. 39-57, 08950 Esplugues de Llobregat, Spain; 6 Centro de Investigación Biomédica en Red de Epidemiología Y Salud Pública (CIBERESP), Instituto de Salud Carlos III, Av. Monforte de Lemos, no. 3-5, pabellón 11, planta 0, 28029 Madrid, Spain; 7 Departament de Cirurgia i Especialitats Medicoquirúrgiques, Facultat de Medicina i Ciències de la Salut, Universitat de Barcelona, Casanova, no. 143, 08036 Barcelona, Spain

**Keywords:** CPAP ventilation, Bronchiolitis, General ward, Pediatric ICU, Safety, Invasive mechanical ventilation

## Abstract

The results of several clinical trials suggest that continuous positive airway pressure (CPAP) for acute bronchiolitis can be more effective than high-flow nasal cannula (HFNC). The use of HFNC involved a minimum reduction (5%) in admissions to the pediatric intensive care unit (PICU) in our hospital. Our main aim was to evaluate its safety and effectiveness as respiratory support for patients with bronchiolitis in a pediatric general ward. A secondary goal was to compare the admissions to PICU and the invasive mechanical ventilation (IMV) rate of patients treated with HFNC and those treated with HFNC/b-CPAP during the 2018–2019 and 2019–2020 epidemic seasons, respectively. Two prospective single-centre observational studies were performed. For the main aim, a cohort study (CS1) was carried out from 1st of November 2019 to 15th of January 2020. Inclusion criteria were children aged up to 3 months with bronchiolitis treated with b-CPAP support when HFNC failed. Epidemiological and clinical parameters were collected before and 60 min after the onset of CPAP and compared between the responder (R) and non-responders (NR) groups. NR was the group that required PICU admission. One hundred fifty-eight patients were admitted to the ward with bronchiolitis and HFNC. Fifty-seven out of one hundred fifty-eight required b-CPAP. No adverse events were observed. Thirty-two out of fifty-seven remained in the general ward (R-group), and 25/57 were admitted to PICU (NR-group). There were statistically significant differences in respiratory rate (RR) and heart rate (HR) between both groups before and after the initiation of b-CPAP, but the multivariable models showed that the main differences were observed after 60 min of therapy (lower HR, RR, BROSJOD score and FiO2 in the R-group). For the secondary aim, another cohort study (CS2) was performed comparing data from a pre-b-CPAP bronchiolitis season (1st of November 2018 to 15th January 2019) and the b-CPAP season (2019–2020). Inclusion criteria in pre-b-CPAP season were children aged up to 3 months admitted to the same general ward with moderate-severe bronchiolitis and with HFNC support. Admissions to PICU during the CPAP season were significantly reduced, without entailing an increase in the rate of IMV.

*Conclusion*: The implementation of b-CPAP for patients with bronchiolitis in a pediatric ward, in whom HFNC fails, is safe and effective and results in a reduction in PICU admissions.

**Table:** 

**What is Known:** *• Bronchiolitis is one of the most frequent respiratory infections in children and one of the leading causes of hospitalization in infants*. *• Several studies suggest that the use of continuous positive airway pressure (CPAP) for acute bronchiolitis can be more effective than the high flow nasal cannula (HFNC). CPAP is a non-invasive ventilation (NIV) therapy used in patients admitted to pediatric intensive care unit (PICU) with progressive moderate-severe bronchiolitis*. *There is little experience in the literature on the use of continuous positive airway pressure (CPAP) for acute bronchiolitis in a general ward*.	
**What is New:** *• CPAP could be safely and effectively used as respiratory support in young infants with moderate-severe bronchiolitis in a general ward and it reduced the rate of patients who required PICU admission*. *• Patients' heart and respiratory rate and their FiO2 needs in the first 60 minutes may help to decide whether or not to continue the CPAP therapy in a general ward*.

**Supplementary Information:**

The online version contains supplementary material available at 10.1007/s00431-022-04616-3.

## Introduction

Bronchiolitis is one of the most frequent respiratory infections in children and one of the main causes of hospitalization in infants, especially during the winter [1–4]. Respiratory support with high-flow nasal cannula (HFNC) has been shown to be a safe and well-tolerated therapy. It reduces the labour of breathing and improves the comfort of patients with moderate bronchiolitis in the general ward [5–9]. Therefore, HFNC support is often used in a pediatric intensive care unit (PICU), and its use is also increasing in general wards [10]. Unfortunately, in these studies [7, 8], HFNC has not shown effectiveness with generalized use. HFNC failure described was around 35% when used in the rescue group receiving low flow. Therefore, it has not demonstrated efficiency or the capacity to reduce the number of admissions to PICU [8, 11]. In our centre, it has led to only a minimum reduction (5%) [12].

Several clinical trials suggest superior effectiveness of the continuous positive airway pressure (CPAP) modality in children with bronchiolitis [13–15]. CPAP is a respiratory therapy used in patients with progressive moderate-severe bronchiolitis [16–18]. Its use reduces their labour in breathing, increases expiratory time and reduces the duration of ventilation and hospital stay [15, 16]. Most children who require CPAP support are referred to PICU. However, recent studies show that treatment with CPAP may be feasible in a general pediatric ward [19, 20]. There are different CPAP devices; of these, the bubble-CPAP (b-CPAP) has a simple assembly and a similar cost to the HFNC device.

The main aim of the study was to evaluate b-CPAP safety as respiratory support for patients with bronchiolitis in a general ward. A secondary goal was to evaluate the effectiveness of the implementation of b-CPAP in a general ward used as a rescue treatment of HFNC failure to reduce PICU admissions.

## Methods

### Study design

Two prospective single-centre observational studies were performed. For the main goal, a cohort study (CS1) was carried out from 1st November 2019 to 15th January 2020. During the 2019–2020 bronchiolitis epidemic season, the use of b-CPAP was implemented within the care protocol for patients with bronchiolitis. The b-CPAP was used as respiratory support for selected patients admitted to a general ward, which offered 20 beds with a nurse: patient ratio 1:6, higher than another hospitalization ward (nurse:patient ratio 1:10). Four b-CPAPs were available simultaneously. The specific care protocol included criteria for initiating and removing CPAP therapy, instructions for setting up the CPAP device and nursing care guidelines. Vital signs were recorded by the nursing staff every hour and every 4 h after stabilization. To prevent complications associated with the nasal mask, pressure sores were assessed every 4 h, the mask support points were modified and a hyperoxygenated fatty acid solution was applied. Medical staff assessed the patient at the start of the treatment, within the first 60 min, and then every 12 h. Vital signs, CPAP parameters (pressure, flow, FiO2), nursing care details and type of feeding were recorded on a specific form.

For the secondary goal, another cohort study (CS2) was performed comparing data from a pre-b-CPAP bronchiolitis season (2018–2019) and the b-CPAP season (2019–2020). Patients included in the pre-b-CPAP cohort were those admitted between 1st of November 2018 and 15th of January 2019 and in the post-b-CPAP period between 1st of November 2019 and 15th of January 2020. Data from the two cohorts were prospectively collected.

These studies were performed in a third-level maternal-child hospital with an average of 600 admissions during the respiratory syncytial virus (RSV) outbreak in the winter season.

### Population

- CS1: Patients included were those aged up to three months admitted to the general ward with a diagnosis of moderate-severe bronchiolitis according to BROSJOD score [21] (0–6 points mild, 7–9 moderate, ≥ 10 severe) and with the indication of b-CPAP support after a failure in the HFNC support.
- CS2: Patients included were also those aged up to three months admitted to the same general ward (with the same nurse:patient ratio) with a diagnosis of moderate-severe bronchiolitis, according to BROSJOD score, and with the indication of HFNC support (and/or b-CPAP in the post-b-CPAP period).
  Informed consent was requested from the family of each patient.

### Indications and setup

The criteria for starting treatment with b-CPAP in infants with bronchiolitis were:

1. Bronchiolitis with BROSJOD score 9–11 points failing on HFNC, meaning those who, 60–90 min after the onset, did not present a score reduction (2 points) nor a significant decrease (> 10 points) in respiratory rate (RR) and/or heart rate (HR).
2. Apnea without bradycardia.

The b-CPAP therapy was contraindicated in infants with a score BROSJOD > 11 points or orofacial malformations.

The device used was b-CPAP (Fisher Paykel®) with a nasal mask. The CPAP pressure was initially set at five cmH2O and progressively increased to a minimum of seven cmH2O [22] with the necessary FiO2 to maintain O2Sat at 93–97%. The O2Sat was continuously monitored by pulse oximetry. Nebulization was not performed during CPAP therapy, but nasal washes were carried out when secretions were copious. Containment measures were applied, such as using a pacifier and administrating sucrose and/or a one-time dose of levomepromazine to achieve good CPAP’s tolerance.

Before the implementation of b-CPAP, specific training on respiratory support in bronchiolitis and the use of this device was given to all healthcare personnel (nurses, nursing assistants and pediatricians). Theory sessions were held to explain the care protocol and the setting of the general ward, combined with a practical simulation on assembling the CPAP device. Clinical cases were presented to resolve questions.

The criteria for starting HFNC in both periods was bronchiolitis with BROSJOD score ≥ 8 points or SatHb < 92% despite the use of 2 L/min with low-flow oxygen cannula.

Those patients with b-CPAP/HNFC support from PICU in the resolution phase of bronchiolitis were excluded from the two studies.

### Outcomes

- CS1: Based on main outcome, two groups are described, the responder (R-group) and non-responder (NR-group). Non-responder was defined as a patient in need of transfer to the PICU in the seven days following the onset of b-CPAP support. The transfer could be made in response to an increase in the BROSJOD score, HR and/or RR, as well as the presence of apnea with hemodynamic instability. Other, secondary, outcomes were length of b-CPAP support (before requiring PICU admission or being weaned) and hospital length of stay (LOS).
- CS2: The main outcomes were the LOS in the general ward before requiring PICU admission, the rates of PICU admission and IMV and the hospital LOS.

### Data collection

- CS1: Epidemiological and clinical variables were collected before the start of CPAP and at 60 min, and comparison was made between the R and NR groups. Complications associated with b-CPAP were considered: pressure sores (irritation, wounds, pressure-induced skin and soft tissue injuries), air leaks (pneumothorax, pneumomediastinum) and aspiration due to vomiting.
- CS2: Data on PICU admission, general ward LOS before PICU admission (if needed), hospital stay and need for IMV were collected. To assess the comparability of the seasons, age of patients and a surrogate of disease severity (the BROSJOD score before the initiation of HFNC) were also collected.

### Statistical analyses

Data comparisons of categorical variables were performed using the Pearson chi-square test or Fisher exact test. Continuous non-normal distributed variables were compared using Mann–Whitney *U* test.

Multivariable analyses were performed using logistic regression models to identify:

- CS1: those variables associated with b-CPAP success or failure (R/NR)
- CS2: the need for PICU admission.

Furthermore, Cox regression models were also used to identify those variables associated with the main time-dependent outcomes (CS1: “CPAP length”; CS2: “length of stay at the general ward before requiring admission to the PICU”).

All the variables related to these outcomes with a cut-off point of *p* < 0.2 in the univariate analyses, as well as other variables which had been found to be associated with respiratory failure in the literature, were considered in the models using the “enter” method. If, approximately, there was more than one predictive variable for every ten outcome events, a second multivariate model was adjusted with the forward method. Collinearity between the variables in each model was assessed using the variance inflation factor (VIF). A VIF < 5 was considered to be not severe enough to require correction.

The Hosmer–Lemeshow test and the − 2 log-likelihood statistic (− 2LL) were used to assess the fit of the models.

In addition, as HR, RR and need for oxygen therapy (FiO2) are included in the BROSJOD score (Supplementary data), alternative models were considered to avoid simultaneously introducing the BROSJOD score with these variables.

All these statistical analyses were performed with SPSS v26.0 software (IBM Corp: Armonk, NY, USA). On the other hand, adjusted survival curves comparing the time-to-failure in the CS2 cohort are shown. To make them, we used the ggadjustedcurves function (survminer package) in R 4.1.3 with the conditional method [23, 24]. Comparisons between the curves of both seasons were done with the log-rank test. A *p*-value < 0.05 was considered as statistically significant.

## Results

### Analysis of the first cohort: CS1 study

One hundred fifty-eight patients with a diagnosis of bronchiolitis required HFNC in the general ward. Fifty-seven of these underwent b-CPAP. 33/57 were males and 54/57 tested positive for RNA detection of RSV in respiratory samples. The main indication (55/57) of b-CPAP was HFNC failure, and only 2/57 showed the presence of apnea (Table 1).

**Table 1:** CS1 study. Comparison of demographic and clinical variables of the responder (R) and the non-responder (NR) groups

		Univariate		
	Total	Responder group (*n* = 32)	Non-responder group (*n* = 25)	*p*-value
**Age** (days)*	39 (25–29)	37 (27–45)	51 (22–66)	0.28**
**Weight** (Kg)*	4.3 (3.5–5)	4.2 (3.5–4.9)	4.6 (3.7–5.6)	0.37**
**Comorbidity** (n)	3	0	3	0.19^a^
**BROSJOD score** (points)*	10 (9–10)	9 (8–10)	10 (9–11)	0.05**
**HR pre-CPAP** (bpm)*	160 (150–175)	155 (144–166)	170 (158–180)	< 0.01**
**RR pre-CPAP** (bpm)*	64 (55–70)	60 (49–66)	65 (60–71)	0.04**
**FiO**_**2**_** pre-CPAP** (%)*	0.34 (0.3–0.4)	34 (29–40)	34 (31–40)	0.33**
BROSJOD score at 60 min*	7 (6–8)	6 (6–7)	8 (7–9)	< 0.01**
**HR at 60 min** (bpm)*	150 (135–160)	145 (135–155)	150 (140–164)	0.04**
**RR at 60 min** (rpm)*	50 (44–60)	45 (40–50)	55 (50–65)	< 0.01**
**FiO**_**2**_** at 60 min** (%)*	32 (30–38)	31 (27–32)	38 (32–40)	< 0.01**
**CPAP length** (hours)*	54 (11–96)	96 (72–120)	10 (3–14)	
**General ward length of stay** (days)*	4 (1–7)	6 (5–8)	1 (1–2)	< 0.01**
**Hospital length of stay** (days)*	9 (7–13)	8 (6–10)	11 (8–19)	< 0.01**

*BROSJOD score*, bronchiolitis score of Sant Joan de Déu, *CI *confidence interval, *HR* heart rate, *OR* odds ratio, *RR* respiratory rate

^*^Median (interquartile range); ^**^Mann–Whitney *U*, ^a^*F*-Fisher

Thirty-two out of fifty-seven (56%) patients treated with b-CPAP remained in the ward (R-group), and 25/57 (44%) were admitted to the PICU (NR-group). Before the beginning of b-CPAP, statistically significant lower HR and RR values were observed in the R-group compared to the NR-group. Moreover, patients in the R-group had significantly lower HR, RR, BROSJOD score and FiO2 after 60 min of therapy, compared to the NR-group (Table 1). In the multivariable models, these parameters before starting b-CPAP lost statistical significance when analysed with the variables at 60 min after b-CPAP support. In these models, HR after 60 min was the main variable associated with NR (Table 2). In the NR-group, the median number of hours of b-CPAP before being admitted to the PICU was 10 (IQR: 3–14). The Cox regression model showed that HR, RR and FiO2 at 60 min after b-CPAP were related with more precocious failure (Table 2). The median general ward and hospital stays were four (IQR: 1–7) and 9 days (IQR: 7–13), respectively.

**Table 2:** CS1 study. Multivariable models (logistic and Cox regressions) with variables associated with the “non-responder” group (b-CPAP failure in the general ward)

	Logistic regression 1*			Cox regression 1*		Logistic regression 2**			Cox regression 2**	
	VIF	Odds ratio (95% CI)	*p*-value	Hazard ratio (95% CI)	*p*-value	VIF	Odds ratio (95% CI)	*p*-value	Hazard ratio (95% CI)	*p*-value
**Age** (months)	1.1	Excluded by forward selection	-	Excluded by forward selection	-	1.1	1.14 (0.46–2.83)	0.78	0.96 (0.51–1.80)	0.89
**BROSJOD scale** (points)	-	-	-	-	-	1.1	1.20 (0.67–2.16)	0.53	1.08 (0.74–1.59)	0.68
**HR pre-CPAP** (bpm)	2.3	Excluded by forward selection	-	Excluded by forward selection	-	-	-	-	-	-
**RR pre-CPAP** (bpm)	2.2	Excluded by forward selection	-	Excluded by forward selection	-	-	-	-	-	-
**BROSJOD Scale at 60 min**	-	-	-	-	-	1.1	2.71 (1.31–5.62)	< 0.01	2.23 (1.59–3.12)	< 0.01
**HR at 60 min** (bpm)	1.9	1.10 (1.01–1.20)	0.02	1.06 (1.02–1.09)	< 0.01	-	-	-	-	-
**RR at 60 min** (rpm)	2.2	1.10 (0.99–1.22)	0.06	1.07 (1.01–1.13)	0.01	-	-	-	-	-
**FiO**_**2**_** at 60 min** (%)	1.4	1.15 (1.00–1.31)	0.05	1.09 (1.01–1.17)	0.02	-	-	-	-	-
		*Hosmer–Lemeshow p-value* = *0.91*		-2LL = 90		*Hosmer–Lemeshow p-value* = *0.33*			-2LL: 129	

*2LL:* − 2 log-likelihood statistic, *BROSJOD score*, bronchiolitis score of Sant Joan de Déu, *CI* confidence interval, *HR* heart rate, *OR* odds ratio, *RR* respiratory rate, *VIF* variance inflation factor

^*^Forward selection of variables; ^**^Enter method of variables

No pressure sores due to the nasal mask, such as pressure-induced skin and soft tissue injuries, were reported, nor were other side effects that would have required the removal of the b-CPAP.

### Analysis of the second cohort: CS2 study

When comparing the pre-b-CPAP season (2018–2019) and the b-CPAP season (2019–2020), the univariant analysis pointed in the direction of reduction in PICU admissions in the second period without changes in IMV rate or hospital stay. There were no differences in age or severity score (BROSJOD) between the seasons (Table 3).

**Table 3:** CS2 study. Comparison of demographic and clinical variables of the pre-CPAP period (2018–2019) and the CPAP period (2019–2020)

	Overall (*n* = 215)	2018–2019 (*n* = 103)	2019–2020 (*n* = 112)	*p*-value
**Age** (months)*	1.6 (0.9–2.4)	1.6 (1.1–2.4)	1.5 (0.8–2.3)	0.49**
**BROSJOD score before HFNC** (points)*	9 (8–10)	9 (8–10)	9 (8–10)	0.83**
**PICU admissions**, *n* (%)	81 (38%)	44 (43%)	37 (33%)	0.14^a^
**General ward stay** (days)*	4 (1–6)	4 (1–6)	5 (2–6)	0.69**
**Hospital stay** (days)*	7 (5–10)	7 (5–10)	6 (5–9)	0.72**
**Invasive mechanical ventilation**, *n* (%)	19 (9%)	7 (7%)	12 (11%)	0.31^a^

*BROSJOD score*, bronchiolitis score of Sant Joan de Déu

^*^Median (interquartile range); **Mann–Whitney *U*; ^a^chi-square

**Fig. 1:**
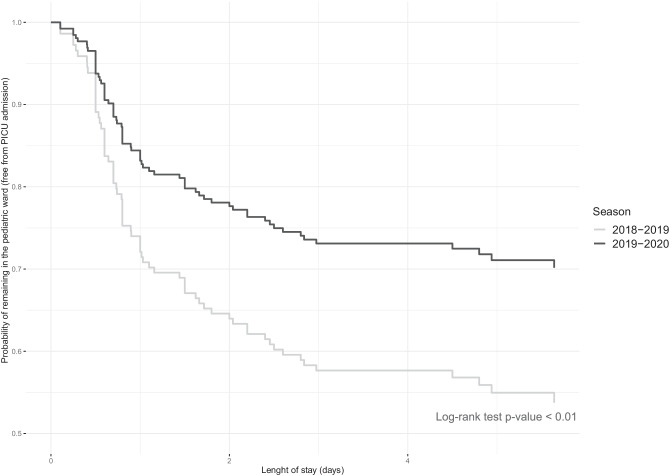
Adjusted survival (time-to-failure) curve for the whole CS2 cohort grouped by season

Controlling for confusion factors, multivariable models revealed that the only statistically significant variables independently associated with the reduction in the need of PICU admission were age and b-CPAP season (Table 4). Adjusted survival curves (by age and BROSJOD score) modelling probability of remain in ward without transfer to PICU for both seasons are shown in Fig. 1.

**Table 4:** CS2 study. Univariate and multivariable logistic and Cox regression models with the outcome event “PICU admission” (2018–2019 and 2019–2020 winter season cohorts)

	Patients who did not required PICU admission (*n* = 134)	Patients who required PICU admission (*n* = 81)	Univariate analysis	Multivariable analysis				
			*p*-value	VIF	Odds ratio (95% CI)	*p*-value	Hazard ratio (95% CI)	*p*-value
**Age** (months)	1.8 (1.2–2.6)*	1.3 (0.7–2.0)*	< 0.01**	1.0	0.51 (0.36–0.73)	< 0.01	0.61 (0.45–0.82)	< 0.01
**BROSJOD score before initiating HFNC** (points)	9 (8–10)*	9 (8–10)*	0.73**	1.0	1.13 (0.90–1.40)	0.29	1.1 (0.9–1.3)	0.21
**Season:** **2018**–**2019,** *n* (%) **2019**–**2020** *n* (%)	59 (44%) 44(55%)	75 (56%) 36 (45%)	0.12^a^	1.0	- 0.44 (0.23–0.82)	0.01	- 0.56 (0.34–0.92)	0.02
					Hosmer–Lemeshow *p* = 0.32		-2LL: 671	

*-2LL:* − 2 log-likelihood statistic, *BROSJOD score*, bronchiolitis score of Sant Joan de Déu, *CI* confidence interval, *HR* heart rate, *OR* odds ratio, *RR* respiratory rate

^*^Median (interquartile range); ** Mann–Whitney *U*; ^a^chi-square

The application of this new care protocol in the general ward was well accepted by the staff due to the standardized practical guidelines and previous specific training.

## Discussion

CPAP therapy in patients with bronchiolitis in PICU has been associated with an improvement in respiratory distress [13, 14, 16, 17], less ventilation time [25] and a reduction in hospital stay [26, 27] and, consequently, in hospital costs. However, a shortage of PICU beds during the winter season is frequent even in high-income countries. For example, in the United Kingdom (UK), there are level 2 high-dependency units (HDU). These units are wards with a higher ratio of nurses to patients, but slightly lower than that in ICU, that attend patients who need more intensive observation, treatment and nursing care than is possible in the general ward. In a critical situation, such as the epidemic RSV season, these units are requested to provide non-invasive ventilation (NIV) to reduce PICU admissions [28].

This study suggests that children with moderate-severe bronchiolitis could be safely treated with NIV such as bubble-CPAP in a hospitalization ward with an optimized nurse-patient ratio. We did not identify any side effects related to its use owing to the theoretical reduction of the provided monitoring with a reduced nurse-patient ratio compared to an HDU or PICU department. Although NIV is applied mostly in PICU, trials supporting NIV in bronchiolitis in general wards are increasing and with favourable results [10, 19, 20, 26], especially in developing countries without PICU facilities [29, 30].

According to our data, the use of CPAP in a general ward as a rescue respiratory support when HFNC failed significantly reduced the number of PICU admissions compared to the previous season. This did not entail an increase in the rate of IMV or a longer hospital stay, as observed by other authors [26, 31]. CPAP therapy could be a valid strategy to optimize resource use and prioritize and select suitable patients in need of PICU care during an epidemic season when an overload situation often occurs. This fact concerns hospital management, and it is reflected in other settings [26, 32, 33].

It should be noted that some studies have demonstrated the superiority of CPAP over HNFCs as pre-emptive respiratory support [13, 27]. However, groups differ about this issue [34]. Therefore, more trials are required to shed further light on the pre-emptive treatment of these patients.

Regarding the failure of CPAP therapy reported in the literature, this usually occurs within the first 6–12 h after the onset of respiratory support [13, 26]. As in previous studies, we observed that this mostly happened during the first 10 h.

Our group found significant differences in respiratory rate between responder and non-responder patients before CPAP support, as was seen in prior studies [13, 16, 18, 26]. Differences were also observed in heart rate between the two groups—something that has not been analysed in previous research. In the first hour with CPAP support, RR, HR, BROSJOD score and FiO2 also differed significantly between responders and non-responders. Taking into account confusion bias by using multivariable statistical models, differences in vital signs before b-CPAP onset lost their statistical significance in favour of differences in the first hour after applying b-CPAP. This could be explained partly either by a very good response to the b-CPAP of patients with high HR and RR or by the dynamic evolution of an initially non-severe bronchiolitis which got worse despite the use of the therapy. As we excluded patients with a BROSJOD score > 11 from receiving b-CPAP therapy in the general ward, our results suggest that it is safe to try b-CPAP support and evaluate these signs in the first 60 min to decide whether or not to continue CPAP therapy in the ward or transferring the patient to PICU.

The main limitation of this study is that it was performed in a general ward with optimized staff and a limited number of patients treated on CPAP, distinct from other hospitalization facilities. Both nursing and medical staff were explicitly trained in CPAP use, and the patient-nurse ratio was higher (1:6) than the average in Spain (1:10). However, in PICUs, more staff are available to treat patients with CPAP support, so that the human factor could play a role in the obtained results. We conducted this study during the first year of implementation of CPAP in the ward. So in the following seasons, the outcomes could improve when staff become more familiar with the CPAP device, its use and improved selection criteria.

In addition, literature is scarce on CPAP support in bronchiolitis in a general ward. So our group mainly compared the results with studies about CPAP use and comparison of CPAP with other therapies (HFNC, invasive ventilation) carried out in PICU, where conditions differ from those in the ward, as previously noted. So until further studies are performed, we would recommend implementing CPAP therapy in a general ward only in those centres where PICU or paediatric transport options are available [19, 32].

Based upon our data and previous studies, we suggest that the pre-emptive use of CPAP as respiratory support in selected patients with bronchiolitis could play a relevant role in a general ward with an optimized nurse-patient ratio [12, 29].

## Conclusion

Our study suggests that bubble-CPAP could be used safely and effectively as respiratory support in young infants with moderate-severe bronchiolitis, in whom HFNC fails, in a general ward. A reduction in PICU admissions was observed after implementing b-CPAP in a paediatric ward, after HFNC failure. The failure of CPAP occurs mostly within the first 10 h, and evaluation at 60 min could ensure its early detection. Higher heart rate and respiratory rate values before b-CPAP can better identify those patients who are best served by bi-level support in the PICU without trying b-CPAP therapy. However, further studies are required to what the optimal respiratory support for these patients is.

## Supplementary information

Below is the link to the electronic supplementary material.

Supplementary file1 (DOCX 16 KB)

## Data Availability

Not applicable.

## Abbreviations

b-CPAP: Bubble continuous positive airway pressure
HR: Heart rate
HFNC: High-flow nasal cannula
IQR: Interquartile range
IMV: Invasive mechanical ventilation
LOS: Length of stay
NIV: Non-invasive ventilation
NR-group: Non-responder group
PICU: Pediatric intensive care unit
RR: Respiratory rate
RSV: Respiratory syncytial virus
R-group: Responder group
