# Supplementary material for: Safety and effectiveness of bubble continuous positive airway pressure as respiratory support for bronchiolitis in a pediatric ward
Source: Eur J Pediatr. 2022 Sep 21;181(12):4039–47. doi: 10.1007/s00431-022-04616-3 (PMC9649485; doi:10.1007/s00431-022-04616-3)
Supplement: Supplementary file 1 — Supplementary file1 (DOCX 16 KB) [file 431_2022_4616_MOESM1_ESM.docx]

**Supplementary data**. BROSJOD score*

| Wheezes/rales | 0: no | | | |
| --- | --- | --- | --- | --- |
|  | 1: expiratory wheezes, inspiratory rales | | | |
|  | 2: expiratory and inspiratory wheezes/rales | | | |
| Indrawing | 0: no | | | |
|  | 1: subcostal, lower intercostal | | | |
|  | 2: previous + supraclavicular + nasal flaring | | | |
|  | 3: previous + upper intercostal + tracheal tug | | | |
| Air entry | 0: normal | | | |
|  | 1: regular and symmetric | | | |
|  | 2: asymmetric | | | |
|  | 3: very reduced | | | |
| Oxygen saturation | Without O_2_ | With O_2_ | | |
|  | 0: >95% |  | | |
|  | 1: 91–94% | 1: >94% with FiO_2_ ≤ 40% | | |
|  | 2: <90% | 2: <94% with FiO_2_ > 40% | | |
| RR (rpm) | 0 | 1 | 2 | 3 |
| <3 m | <40 | 40–60 | 60–70 | >70 |
| 3–12 m | <30 | 30–50 | 50–60 | >60 |
| 12–24 m | <30 | 30–40 | 40–50 | >50 |
| HR (bpm) |  |  |  |  |
| <1 year | <130 | 130–150 | 150–170 | >170 |
| 1–2 years | <110 | 110–120 | 120–140 | >140 |

O2, oxygen; FiO2, fraction of inspired oxygen; RR, respiratory rate; HR, heart rate; rpm, respirations per minute; bpm, beats per minute.

* Balaguer M, Alejandre C, Vila D, Esteban E, Carrasco JL, Cambra FJ, Jordan I. Bronchiolitis Score of Sant Joan de Déu: BROSJOD Score, validation and usefulness. Pediatr Pulmonol. 2017;52(4):533-539. doi:10.1002/ppul.23546
